# Immune regulatory mechanisms in the tumor microenvironment and their applications in cancer therapy: from basic research to clinical translation

**DOI:** 10.3389/fimmu.2026.1810065

**Published:** 2026-05-29

**Authors:** Yafei Feng, Yu Wang, Xueyi Li, Yuze Wang, Ming Zhang

**Affiliations:** 1 The Second Hospital, Dalian Medical University, Dalian, Liaoning, China; 2 Department of Pharmacy, The Second Hospital, Dalian Medical University, Dalian, Liaoning, China; 3 Department of Cardiology, The Second Hospital, Dalian Medical University, Dalian, Liaoning, China; 4 Department of Respiratory Medicine, The Second Hospital, Dalian Medical University, Dalian, Liaoning, China; 5 Department of General Surgery, The Second Hospital, Dalian Medical University, Dalian, Liaoning, China

**Keywords:** adoptive cell therapy, cancer immunotherapy, clinical translation, immune checkpoint inhibitors (ICIs), metabolic reprogramming, myeloid-derived suppressor cells (MDSCs), therapeutic resistance, tumor microenvironment (TME)

## Abstract

Cancer immunotherapy has significantly advanced oncological care, offering durable responses for a subset of patients. However, the efficacy of these treatments is frequently hindered by primary and acquired resistance orchestrated by the tumor microenvironment (TME). Within the highly heterogeneous TME, multidirectional interactions between tumor cells, stromal components, and infiltrating immune cells actively suppress anti-tumor immunity. This review comprehensively examines the multifaceted immune regulatory mechanisms within the TME, with a specific focus on the immunosuppressive roles of tumor-associated macrophages (TAMs), myeloid-derived suppressor cells (MDSCs), and regulatory T cells (Tregs). We further explore how metabolic reprogramming—such as the Warburg effect, lactate accumulation, and hypoxia—creates a hostile niche that impairs effector T cell function and promotes immune evasion. Bridging basic research to clinical translation, we systematically evaluate current TME-targeted therapeutic strategies, including immune checkpoint inhibitors (ICIs), adoptive cell therapies, oncolytic viruses, and anti-angiogenic agents. The review also highlights emerging combinatorial approaches designed to remodel the immunosuppressive stroma and normalize the tumor vasculature. Finally, we discuss critical challenges in clinical application, such as tumor heterogeneity and the need for predictive biomarkers, emphasizing that personalized strategies tailored to specific TME phenotypes are essential for overcoming therapeutic resistance and improving patient outcomes.

## 1 Introduction

Cancer, a leading global cause of mortality, is characterized by cardinal hallmarks including unregulated cell proliferation, invasive growth and metastatic dissemination (1). Conventional cancer therapies (such as surgery, chemotherapy and radiotherapy) and precision strategies have significantly advanced clinical management. In recent decades, cancer immunotherapy has emerged as an effective therapeutic paradigm in oncology, reshaping cancer treatment of malignant tumors (2, 3). Immune checkpoint inhibitors (ICIs), chimeric antigen receptor T (CAR-T) cell therapy, personalized therapeutic cancer vaccines and oncolytic viruses have achieved remarkable clinical breakthroughs, eliciting durable and long-term anti-tumor responses in a subset of patients (4). Nonetheless, these therapeutic approaches face significant limitations, including primary or acquired drug resistance, severe off-target toxicities to normal tissues, and the persistence of minimal residual disease (5). These issues inevitably lead to treatment failure and tumor recurrence, leaving plenty of critical unmet clinical needs in the oncology field that demand innovative solutions (6).

However, the failures are not merely tumor-intrinsic, but are largely orchestrated by immune regulatory circuits embedded in the tumor microenvironment (TME). As a highly dynamic, complex, and plastic cellular and molecular ecosystem enveloping tumor cells, the TME acts as the indispensable regulator of tumorigenesis, malignant progression, metastasis, and therapeutic response across all stages (7). Over the past several years, recent translational and basic research has elucidated how the TME drives immune evasion and tolerance, leading to the failure of anti-tumor immunity. Key mechanisms undermining anti-tumor immunity include regulatory T cells (Tregs), M2-polarized tumor-associated macrophages (TAMs), and myeloid-derived suppressor cells (MDSCs) (8). Additionally, immune checkpoint molecules exhibit aberrant upregulation and their signaling pathways are activated (9). Moreover, the TME undergoes metabolic reprogramming, creating a nutrient-deprived and immunosuppressive metabolic niche (9). This metabolic shift directly impairs the proliferation, activation, and effector functions of anti-tumor effector T cells, while also compromising professional antigen-presenting cell function and antigen presentation capabilities (10, 11). Currently, targeted modulation of TME has been widely recognized as a potential therapeutic approach for novel cancer immunotherapy strategies. These insights have driven the development of TME-targeted therapies and rational combination regimens (12, 13).

This review provides a comprehensive summary of the major cellular and molecular constituents of the TME, with a particular emphasis on the multidirectional regulatory roles of distinct immune cell populations in shaping the TME phenotype and tumor fate. We discuss recent advances in elucidating the TME immune regulatory mechanisms, and examine their critical roles in driving malignant tumor progression and therapeutic resistance. Furthermore, we highlight their translational applications in developing novel TME-targeted immunotherapies and rational combination treatment strategies. Meanwhile, we critically address the major challenges in bridging basic TME research and clinical translation, ultimately aiming to improve overall cancer treatment outcomes. Finally, we discuss the open questions and outstanding obstacles in translating TME biology into clinical practice.

## 2 Composition and characteristics of the TME

### 2.1 Tumor cells and immune-tolerant TME

A key hallmark of malignant tumor cells is their capacity to create an immune-tolerant TME. This is achieved by secreting immunosuppressive cytokines such as transforming growth factor-β (TGF-β) and interleukin (IL)-10 and by recruiting immunosuppressive cells, including MDSCs and Tregs (14, 15). At the cell-surface level, tumor cells modulate immune checkpoint pathways—most notably by upregulating programmed death-ligand 1 (PD-L1), which binds to programmed cell death protein-1 (PD-1) on T cells and suppresses their activation and effector function (16, 17). Moreover, tumor cells frequently downregulate major histocompatibility complex (MHC) class I molecules and antigen-processing machinery, impairing antigen presentation and enabling escape from cytotoxic T lymphocyte (CTL) recognition (18). Metabolic reprogramming of tumor cells further reinforces this immunosuppressive milieu: heightened aerobic glycolysis leads to the accumulation of lactate and the depletion of key nutrients in the TME, thereby compromising immune cell function (19, 20).

### 2.2 Immune cells in the TME

**Figure 1:**
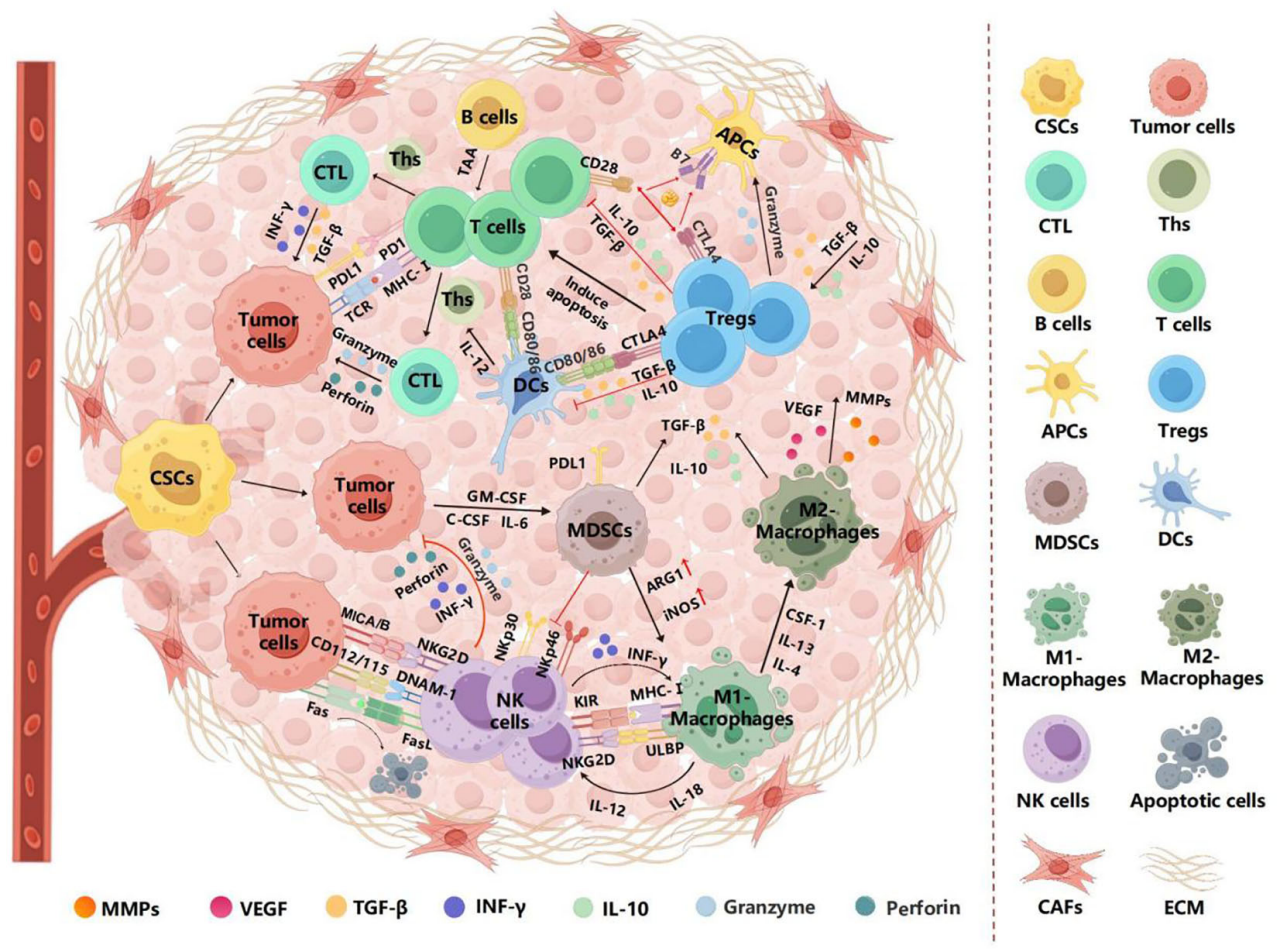
Schematic overview of immune cells in the TME. Tumor cells reside in a dynamic microenvironment composed of various immune cells and stromal elements. Multiple immunoregulatory signals—including PD-1/PD-L1 checkpoint interactions, immunosuppressive cytokines, and metabolic by-products foster an environment conducive to tumor growth and immune evasion. TME, tumor microenvironment. CSCs, cancer stem cells. CTL, CD8^+^ cytotoxic T lymphocyte. Ths, CD4^+^ helper T cells. APCs, antigen-presenting cells. Tregs, regulatory T cells. MDSCs, myeloid-derived suppressor cells. DCs, dendritic cells. NK cells, natural killer cells. CAFs, cancer-associated fibroblasts. ECM, extracellular matrix. MHC-I, major histocompatibility complex Class I. TAA, tumor-associated antigen. VEGF, vascular endothelial growth factor. MMPs, matrix metalloproteinases. Created in BioRender. Zhang, M. (2026) https://BioRender.com/1vdt2hv.

The TME hosts a wide array of immune cells, including T cells, B cells, natural killer cells (NK cells), TAMs, dendritic cells (DCs), MDSCs and Tregs (21) (**Figure 1**). The quantity, phenotype and localization of these cells can differ significantly among tumor types or even within different areas of the same tumor, leading to varied immune responses—either anti-tumor or pro-tumor (22). This phenomenon of immune “polarity” underscores the necessity to discern specific cell populations and signals that orchestrate the TME (23).

#### 2.2.1 TAMs

Macrophages are central players in the innate immune system and, when present in the TME, are termed TAMs. TAMs are generally classified along an M1 (pro-inflammatory, anti-tumor) to M2 (anti-inflammatory, pro-tumor) polarization spectrum. M1 macrophages secrete pro-inflammatory cytokines (TNF-α, IL-1β, IL-12) that recruit immune cells and mediate tumor killing, whereas M2 macrophages promote tumor progression through immunosuppressive cytokines (IL-10, TGF-β), angiogenesis, extracellular matrix (ECM) remodeling, and suppression of tumor-infiltrating lymphocytes (TILs) activity (24–27).

Tumor-derived factors, such as colony-stimulating factor 1 (CSF-1), IL-4, and IL-13, frequently skew macrophages from the M1 phenotype toward M2 macrophages (28, 29). Interferon-γ (IFN-γ) secreted by NK cells serves as a key factor in inducing M1 polarization, establishing a positive feedback loop wherein M1 macrophage-derived IL-12 stimulates NK cells to produce IFN-γ, which subsequently acts back on macrophages to further augment IL-12 secretion (30, 31).

TAM reprogramming in cancer is driven by multiple signaling pathways, among which IL-6/STAT3 represents a particularly well-characterized, cross-cancer mechanism. In glioblastoma, for example, tumor cell-secreted IL-6 activates the STAT3 signaling pathway in both monocyte-derived macrophages and resident microglia, promoting M2 polarization (32, 33). Analogous IL-6/STAT3-mediated M2 polarization has been documented in breast cancer, hepatocellular carcinoma, and pancreatic cancer, suggesting that this represents a common mechanism of TAM reprogramming across diverse tumor types (34–36). Other signaling pathways, including NF-κB, Notch, and PI3K-Akt, similarly contribute to macrophage polarization in a context-dependent manner (37).

#### 2.2.2 Tumor-infiltrating immune cells

##### 2.2.2.1 T lymphocytes

T lymphocytes are central effectors of antitumor immunity, with CD8^+^ T cells acting as the primary cytotoxic mediators and CD4^+^ helper T (Th) cells supporting their activation and function. Upon recognition of tumor-associated antigens (TAAs) presented by MHC-I, CD8^+^ T cells differentiate into CTLs. These CTLs eliminate tumor cells through the secretion of effector molecules such as IFN-γ, perforin, and granzymes, ultimately inducing tumor cell apoptosis (38, 39).

However, within the TME, persistent antigen exposure and immunosuppressive signals frequently drive CD8^+^ T cells into an exhausted state. This state is characterized by impaired effector function and sustained expression of inhibitory receptors, including PD-1 (40). Tumor cells often upregulate PD-L1, which interacts with PD-1 on T cells to suppress T cell receptor (TCR) signaling, promote immune tolerance, and facilitate tumor progression (41).

Immune checkpoint blockade therapies targeting the PD-1/PD-L1 axis have demonstrated remarkable clinical efficacy in malignancies such as melanoma and non-small-cell lung cancer (NSCLC) by partially restoring T cell function (42). Moreover, combination strategies targeting multiple inhibitory pathways—including PD-1/PD-L1, Cytotoxic T-lymphocyte-associated protein 4 (CTLA-4), Lymphocyte-activation gene 3 (LAG-3), and T cell immunoglobulin domain and mucin domain-3 (TIM-3) —are emerging as promising approaches to overcome T cell exhaustion more effectively (43).

Beyond PD-1/PD-L1, CTLA-4, LAG-3, and TIM-3, several novel strategies are emerging to combat T cell exhaustion. T cell immune receptor with Ig and ITIM domains (TIGIT), which competes with the co-stimulatory receptor CD226 for binding to CD155, represents a promising next-generation checkpoint target (44). In the randomized phase II CITYSCAPE trial, tiragolumab plus atezolizumab improved objective response rate (ORR) and progression-free survival (PFS) over atezolizumab alone in PD-L1 high NSCLC, supporting further clinical development of TIGIT blockade (45). Inhibitors of the ectonucleotidases CD39 and CD73 disrupt the adenosinergic axis, in which hypoxia-driven adenosine engages A2A receptors to suppress tumor-infiltrating T cells (46). T cell–engaging bispecific antibodies bridge tumor antigens and CD3 to deliver MHC-independent activating signals at the tumor site, bypassing defective endogenous TCR signaling (47). Finally, DNA methyltransferase inhibitors such as decitabine can reshape the epigenetic landscape of exhausted T cells, restoring their proliferative and effector capacities (48).

In addition to checkpoint inhibition, adoptive T cell therapy (ACT) offers a powerful strategy for precision cancer immunotherapy. Among ACT modalities, CAR-T cell therapy involves the genetic engineering of T cells to express synthetic receptors that recognize specific TAAs, thereby enhancing tumor targeting and cytotoxicity. CAR-T therapy has achieved substantial success in hematological malignancies and continues to be optimized for application in solid tumors (49).

CD4^+^ Th cells play a pivotal role in orchestrating antitumor immune responses by producing cytokines that enhance the activity of CD8^+^ T cells and other immune populations. Upon activation, Th cells differentiate into distinct functional subsets characterized by specific cytokine profiles and effector functions. Among these subsets, Th1 cells are generally associated with antitumor immunity through the secretion of IFN-γ, which promotes CD8^+^ T cell activation and enhances effector immune cell infiltration within the TME (50).

In contrast, the TME is frequently biased toward Th2 or Th17 polarization, which may facilitate tumor progression by promoting angiogenesis, immune suppression, and tolerance. Th17 cells, in particular, exhibit context-dependent functions in cancer. In hepatocellular carcinoma, Th17 cells can exacerbate tumor development and liver injury through the production of IL-17 and IL-22 (51). The impact of Th17 cells in cancer is highly context-dependent, as Th17-mediated inflammation may either enhance antitumor immunity or, under conditions of chronic activation, contribute to tumor progression and metastasis (52).

Tregs are essential for maintaining immune homeostasis; however, their accumulation within the TME promotes immune evasion. Tregs suppress CTL activity through the secretion of immunosuppressive cytokines, including IL-10, TGF-β, and IL-35. Furthermore, Tregs suppress effector T cell function through high-affinity IL-2 consumption and under certain conditions may also induce apoptosis of effector T cells (53, 54). A central mechanism of Treg-mediated suppression involves CTLA-4, which competes with CD28 for binding to B7 molecules (CD80/CD86) on antigen-presenting cells (APCs), thereby limiting costimulatory signaling and dampening T cell activation (55, 56). In addition, Tregs can modulate DCs function through CTLA-4–dependent interactions, impairing antigen presentation and promoting immune tolerance. Beyond CTLA-4, inhibitory receptors such as LAG-3, a CD4 homolog with high affinity for MHC class II (MHC-II) molecules, further contribute to immune suppression by regulating T cell activation and influencing DC activity within the TME (57–59).

##### 2.2.2.2 B lymphocytes

Within the TME, B lymphocytes comprise functionally distinct subsets, including naïve B cells, memory B cells, antibody-secreting plasma cells, and regulatory B cells (Bregs) (60, 61). B cells can function as professional APCs, internalizing TAAs and presenting them to T cells to enhance CTL activation. Plasma cells and memory B cells support anti-tumor humoral responses through antibody-dependent cellular cytotoxicity (ADCC) and immune complex formation. However, B cells can also adopt immunosuppressive phenotypes: Bregs, characterized by IL-10 and TGF-β production, promote expansion of Tregs and MDSCs, dampening the CTL response (61, 62). This functional plasticity of B cells—acting as both tumor promoters and suppressors depending on local immune context—is a key factor in cancer progression. In tumors with well-developed tertiary lymphoid structures (TLS) containing germinal centers, B cells generally support anti-tumor immunity, whereas in tumors lacking TLS, B cells may adopt regulatory roles that dampen immune responses (63, 64).

##### 2.2.2.3 DCs

DCs are essential APCs in the TME, playing a crucial role in initiating adaptive immune responses. DCs capture and process tumor antigens through their specialized phagocytic and endocytic machinery, and subsequently present processed peptides on MHC molecules to prime T cells (65). Upon encountering pathogens or tumor antigens, DCs undergo maturation and express high levels of co-stimulatory molecules, such as CD80 and CD86 (65, 66). These molecules engage with CD28 on T cells, promoting T cell activation and survival. In addition, DCs secrete cytokines like IL-12 that help drive Th1 cell differentiation, fostering strong cellular immunity. They present these antigens to naive T cells, promoting their aggregation, proliferation, and supporting the survival of effector T cells at tumor sites (65).

However, the TME impacts DC differentiation and maturation, leading to a reduction in antigen cross-presentation and the downregulated expression of co-stimulatory molecules. Consequently, DCs may acquire immunosuppressive or immunotolerant phenotypes, a process driven by diverse factors in the TME, including immunosuppressive cytokines and immune checkpoint molecules (67).

Tregs suppress DC maturation and function through both direct cell-to-cell interactions and the secretion of inhibitory cytokines, such as IL-10 and TGF-β. This interaction helps maintain immune tolerance but also contributes to immune evasion in tumors (68). Moreover, Tregs are capable of inhibiting DC-mediated antigen presentation, thereby impairing the activation of anti-tumor T cells (69).

In hepatocellular carcinoma patients, the proportion of DCs in local lymph nodes is often reduced, and the number of functional DCs is significantly decreased. Studies have shown that tumor-derived exosomes inhibit the expression of surface maturation-related molecules on DCs, dampen cytokine secretion, and impair their ability to stimulate the proliferation of allogeneic T lymphocytes. This inhibition of DC maturation within tumors is considered a primary reason for the suboptimal clinical outcomes observed in antitumor immunotherapy (70).

##### 2.2.2.4 NK cells

NK cells occupy a prominent position in the innate immune system, mainly responsible for detecting and eliminating virus-infected cells and tumor cells. Unlike T cells, NK cells do not require antigen presentation via MHC molecules for activation, which enables them to respond quickly to a wide range of infections and malignancies (71). They recognize stressed, infected or transformed cells through a balance of activating and inhibitory receptors, such as Natural Killer Group 2D (NKG2D) and Killer cell immunoglobulin-like receptors (KIRs), and kill target cells through the release of cytotoxic molecules like perforin and granzymes (72).

The activating receptor NKG2D on NK cells recognizes stress-induced ligands including MHC-I polypeptide-related sequence A/B (MICA/B) and UL16-binding protein (ULBP) family proteins on tumor-transformed cells, enhancing NK cell cytotoxicity. Meanwhile, MHC-I molecules regulate NK cell activity through inhibitory receptors such as KIRs, preventing excessive immune reactions (73, 74). NK cell dysfunction within the TME is a significant barrier to successful cancer immunotherapy. Despite their potential to exert potent antitumor effects by directly killing tumor cells and producing cytokine IFN-γ, which helps modulate the immune response, tumors often develop mechanisms to evade NK cell recognition, such as downregulating MHC-I or secreting immunosuppressive cytokines like TGF-β (75).

Furthermore, cytokines and chemokines in the TME can reduce the density of cytotoxic NK cells, increase the density of immature NK cells, and alter their phenotype, transforming them into tumor-associated cells (76). Tumor cells express stress-induced ligands, such as NKG2D ligands (MICA/B and ULBP) and DNAX Accessory Molecule-1 (DNAM-1) ligands (CD112 and CD155), which activate NK cells through the activating receptors NKG2D and DNAM-1, respectively (77). These ligands are upregulated on tumor cells in response to stress, including DNA damage, hypoxia, or oncogene activation, and serve as “danger signals” that alert NK cells to the presence of abnormal cells (78). Furthermore, NK cells express FasL, which binds to Fas on tumor cells and induces programmed cell death (apoptosis) via the extrinsic apoptotic pathway (79, 80). Recent studies have focused on enhancing NK cell activity through immune checkpoint inhibition or NK cell-based adoptive therapy, demonstrating promising preclinical and clinical results in cancers like leukemia, lymphoma and solid tumors (81–83).

Glioblastoma multiforme (GBM), a prototypical immunologically “cold” tumor, presents unique challenges for NK cell–mediated immunity. The Central Nervous System (CNS) location constitutes an immune-privileged sanctuary site, where the blood–brain barrier (BBB) severely restricts immune cell penetration (35). Within the GBM microenvironment, NK cells exhibit a paradoxical dual role: while they retain intrinsic cytotoxic capacity through NKG2D-mediated recognition of stress-induced ligands such as MICA/B and ULBPs on GBM cells, tumor-derived TGF-β, IL-6, and prostaglandin E2 (PGE2) profoundly suppress NK cell activation and downregulate activating receptors including NKG2D (84, 85). To overcome these barriers, ex vivo–expanded and IL-15–stimulated NK cells delivered locoregionally via stereotactic intratumoral or intracavitary injection have shown promising preclinical and early clinical activity, while focused ultrasound–mediated BBB opening is being explored as a complementary strategy to enhance the intratumoral delivery of immunotherapeutic agents in GBM (86, 87). Furthermore, ErbB2 (HER2)–specific chimeric antigen receptor natural killer (CAR-NK) cells (NK-92/5.28.z) have demonstrated potent antitumor activity in orthotopic GBM xenograft and syngeneic mouse models, providing the preclinical rationale for the CAR2BRAIN phase I trial (NCT03383978), which has completed its dose-escalation phase and demonstrated safety of intracerebral NK-92/5.28.z administration in recurrent HER2-positive glioblastoma (88, 89).

#### 2.2.3 MDSCs

MDSCs represent an immunosuppressive population that expands in pathological conditions like cancer, driven by tumor-derived cytokines such as granulocyte-macrophage colony-stimulating factor (GM-CSF), IL-6, and granulocyte colony-stimulating factor (G-CSF) (90). MDSCs include two main subtypes: granulocytic (G-MDSCs) and monocytic (M-MDSCs) (91). These cells exert potent immunosuppressive effects through upregulation of arginase-1 (ARG1) and inducible nitric oxide synthase (iNOS), and have crosstalk with Tregs and macrophages simultaneously (92, 93). The main mechanisms of MDSC-mediated immunosuppression include expressing immune checkpoint molecules such as PD-L1 (94), consuming the amino acids necessary for T cell function (94), generating reactive oxygen species (ROS) and reactive nitrogen species (RNS) (95), secreting the immunosuppressive cytokines TGF-β and IL-10 (96), producing extracellular adenosine through CD39/CD73 ectonucleotidases (97), and downregulating the expression of NK cell surface activation receptors NKp30, NKG2D, and NKp46, thereby mediating the functional inhibition of NK cells (98). In addition, MDSCs can respond to various driving signals and subsequently migrate to lymphoid tissue, tumor lesions, and potential pre-metastatic niches. Various chemokines secreted by tumor cells, such as CCL2, CCL5, CXCL5, and CXCL12, play a crucial role in attracting MDSCs to traverse the vascular barrier (99).

### 2.3 Non-immune cells and the ECM

**Figure 2:**
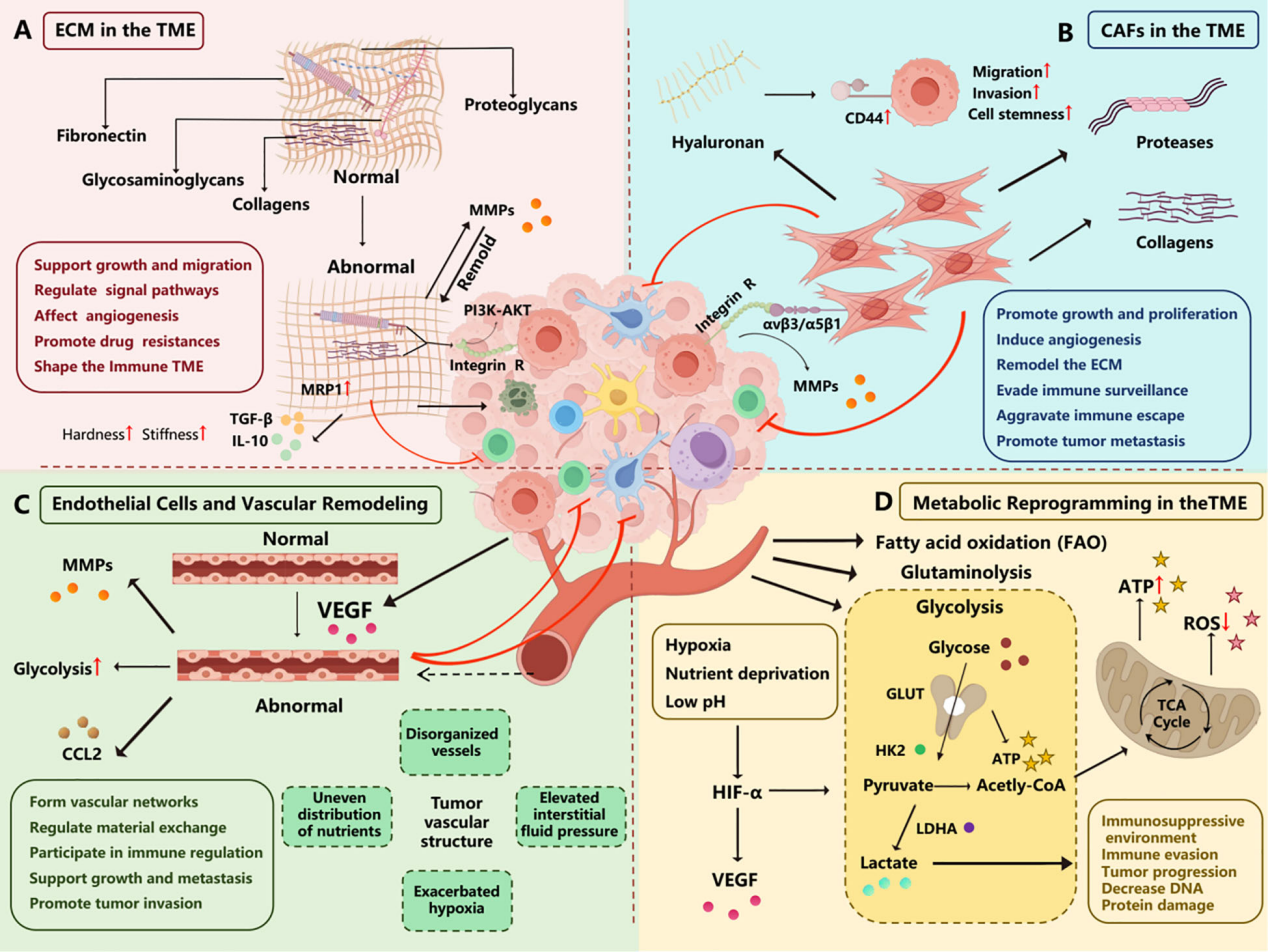
Key non-immune components of the TME. **(A)** ECM remodeling. In contrast to the physiologically organized ECM composed of fibronectin, collagens, proteoglycans, and glycosaminoglycans, the tumor ECM becomes densely remodeled with elevated collagen and fibronectin deposition and increased matrix stiffness. MMP-mediated proteolysis, integrin–PI3K–AKT signaling, and TGF-β/IL-10 release collectively sustain this aberrant architecture, facilitating tumor growth, angiogenesis, drug resistance, and immune exclusion. **(B)** CAFs. Activated CAFs upregulate CD44 and acquire enhanced migratory and invasive capacity. Through secretion of MMPs, hyaluronan, and collagens, CAFs remodel the ECM and promote tumor proliferation, angiogenesis, immune evasion, and metastasis. **(C)** Vascular remodeling. Dysregulated VEGF signaling and MMP activity drive the transition from organized vasculature to tortuous, leaky tumor vessels. Concurrent endothelial glycolytic reprogramming and CCL2 secretion contribute to uneven perfusion, elevated interstitial fluid pressure, and intratumoral hypoxia, further supporting invasion and metastasis. **(D)** Metabolic reprogramming. Under hypoxic, nutrient-deprived, and acidic conditions, HIF-1α orchestrates enhanced aerobic glycolysis, fatty acid oxidation, and glutaminolysis. Accumulation of lactate and other metabolites generates an immunosuppressive milieu that impairs effector immune function and facilitates tumor progression. Created in BioRender. Zhang, M. (2026) https://BioRender.com/ckaxr1j.

Besides immune components, non-immune cells and ECM components also play crucial roles in modulating immune cell functions and shaping the overall immune response within tumors (100). Non-immune cells such as cancer-associated fibroblasts (CAFs), endothelial cells, and adipocytes interact with ECM components to create a microenvironment that can either promote or inhibit immune cell activity, thereby contributing to tumor progression and immune evasion (101) **Figure 2**.

#### 2.3.1 ECM remodeling and TME modulation

The ECM is a core non-cellular component of the TME, comprising collagens, fibronectin, proteoglycans, and glycosaminoglycans (102, 103). In normal tissues, the ECM provides structural and biochemical cues to guide cell adhesion and motility. However, in the TME, the ECM not only provides structural support for tumor cells but also plays an integral role in modulating immune cell behavior through aberrant deposition and remodeling. ECM stiffness significantly affects the infiltration and activity of immune cells. Elevated stiffness and interstitial pressure can enhance tumor cell invasion and, paradoxically, hinder immune effector cells from penetrating the tumors (104, 105). T cells in soft matrix environments possess higher migration ability, while ECM stiffness can also affect the metabolism and activation status of immune cells (106). Besides, high ECM stiffness promotes macrophage polarization toward the immunosuppressive M2 phenotype, increasing secretion of cytokines such as TGF-β and IL-10, thereby inhibiting T cell function and promoting tumor immune escape (107, 108). ECM components such as collagen or fibronectin can activate intracellular signaling pathways (such as PI3K-AKT and MAPK) by binding to integrin receptors on the surface of tumor cells, thereby promoting the expression of PD-L1 in tumor cells (109). Furthermore, the hypoxic tumor microenvironment can induce the upregulated expression of ECM components such as fibronectin, promote endothelial cell migration and angiogenesis, and maintain tumor nutrition supply (110).

Additionally, the ECM is remodeled through the actions of matrix metalloproteinases (MMPs), which modulate immune cell migration by degrading ECM components. This dynamic remodeling creates an environment that can either promote or hinder immune cell access to tumor sites, depending on the interplay between immune cells and ECM components (111).

Furthermore, increased ECM stiffness can overactivate integrin signaling pathways within tumor cells by regulating cell survival and anti-apoptotic protein expression through downstream molecules such as Focal adhesion kinase (FAK), thereby leading to a decrease in the efficiency of drug-induced cell apoptosis and promoting the development of drug resistance (112–114).

#### 2.3.2 CAFs

As one of the most abundant non-immune cell types in the TME, CAFs are paramount mediators of ECM remodeling, secreting collagens, hyaluronan, and proteases that shape the architecture of the TME. Studies have demonstrated that CAFs, through their secretion of ECM proteins, create physical barriers that prevent the infiltration of immune cells, such as T cells and DCs, into the tumor core, thereby exacerbating the immunosuppressive nature of the TME and contributing to immune escape (115, 116).

Integrins are a type of cell surface receptor that mediate cell adhesion to the ECM and participate in cell migration, proliferation, and signal transduction. Highly expressed integrins such as αvβ3 and α5β1 enable CAFs to reshape the matrix structure by binding to fibronectin and collagen in the ECM, forming dense fibrous tissue that physically blocks immune cell penetration (117, 118). CAFs also secrete multiple immunosuppressive cytokines, including TGF-β, IL-6, and CXCL12, which suppress the anti-tumor function of T cells and NK cells and promote the recruitment and activation of MDSCs and Tregs (119, 120). In some tumors, CAFs are classified into pro-inflammatory CAFs (iCAFs) and myofibroblastic CAFs (myCAFs), with their functional properties being driven by different signaling pathways (121). In pancreatic cancer, iCAFs produce IL-6, CXCL1 and other pro-inflammatory cytokines that recruit immunosuppressive cells, while myCAFs produce ECM proteins such as type I collagen and fibronectin that directly form a physical barrier (122, 123).

#### 2.3.3 Endothelial cells and vascular remodeling

As the layer of cells on the inner wall of blood vessels, endothelial cells are of critical importance in regulating immune cell trafficking. During the growth process, tumor cells secrete vascular endothelial growth factor (VEGF) and other angiogenic factors in order to obtain more nutrients and oxygen to support their proliferation and spread, which stimulate endothelial cell proliferation and migration (124). Nevertheless, the newly formed tumor vasculature is often structurally abnormal with disorganized vessels, which combined with elevated interstitial fluid pressure, result in limiting the infiltration of immune cells into the tumor core and exacerbating hypoxia, ultimately constructing an abnormal tumor vascular network (125). Moreover, VEGF dampens DC maturation and T-cell activation while simultaneously recruiting MDSCs and Tregs to the TME, further promoting an immunosuppressive environment (126). Notably, Tumor Endothelial Cells (TECs) can express immune checkpoint molecules such as PD-L1, directly inhibiting T cell activation and recruiting inhibitory immune cells like MDSCs by secreting chemokines, such as CCL2 simultaneously (96, 127). In addition, abnormal blood vessels lead to uneven distribution of nutrients and oxygen; as such, tumor cells are able to adapt to the hypoxic microenvironment through glycolysis, while releasing metabolites such as lactate to maintain an acidic environment, promote activation of MMPs and degrade ECM for metastasis (128–130).

### 2.4 Metabolic reprogramming in the TME and its impact on immune responses

A key feature of TME is metabolic reprogramming, which enables tumor cells to accommodate the hostile conditions such as hypoxia, nutrient deprivation and oxidative stress (131). A hallmark of tumor cells is the Warburg effect, a preference for glycolysis even under aerobic conditions, leading to the accumulation of lactate and other acidic metabolites. Additionally, tumors commonly rewire pathways for glutamine or fatty acid utilization (132).

#### 2.4.1 The Warburg effect

The Warburg effect, or aerobic glycolysis, remains a cornerstone in cancer metabolism. Cancer cells rely heavily on glycolysis for rapid biomass production, even in the presence of oxygen (133). Moreover, researchers have found that oncogene-driven tumors, including those with PI3K/Akt/mTOR activation, can upregulate glycolytic enzymes such as hexokinase 2 (HK2) (134, 135), further enabling rapid ATP production and anabolic reactions. Studies have compellingly confirmed that tumor cells with high glycolytic activity can outcompete adjacent cells for glucose in the TME, creating nutrient competition and accelerating tumor progression (19, 136). The preference for glycolysis allows tumor cells to generate ATP quickly, albeit in smaller amounts compared with Oxidative Phosphorylation (OXPHOS), while providing metabolic intermediates necessary for biosynthesis, such as nucleotides, lipids, and amino acids (132, 137).

#### 2.4.2 Warburg effect and TME

One of the major drivers of the Warburg effect in tumors is the TME, often characterized by hypoxia, nutrient deprivation, and low pH. Rapid tumor growth can outpace local angiogenesis, forming a hypoxic microenvironment (138, 139). Hypoxia activates Hypoxia-inducible factor 1-alpha (HIF-1α), which then promotes the expression of glucose transporters (such as GLUT1) and glycolytic enzymes (including lactate dehydrogenase A and pyruvate kinase M2), thereby enhancing glycolytic capacity to meet the energy needs of tumor cells (140–142). Angiogenesis can be induced by HIF-1α-mediated upregulation of VEGF, providing nutrition and oxygen to the tumor (143). Lactate, once considered merely a waste product of glycolysis, contributes to immune evasion and tumor progression by creating an immunosuppressive acidic microenvironment (20, 144, 145). Tumor cells secrete lactate as a byproduct of glycolysis, which is then taken up by surrounding stromal cells, such as CAFs or endothelial cells, to fuel mitochondrial oxidative phosphorylation in what is known as the “reverse Warburg effect” or “lactate shuttle” (146, 147). This metabolic symbiosis within the TME facilitates rapid tumor progression. Additionally, lactate modulates immune cell activity, fostering an environment conducive to tumor survival and metastasis (148, 149). Acidic conditions inhibit the activity of cytotoxic T cells and NK cells, weakening immune surveillance, while promoting the recruitment and function of Tregs and MDSCs, which further suppress immune responses (150–152). Additionally, TAMs can adopt a glycolytic metabolism that supports their pro-tumorigenic M2 polarization, promoting angiogenesis and immune evasion (153, 154). High glucose consumption by tumor cells through increased glycolysis leads to glucose depletion in the TME, limiting the function of immune cells due to insufficient energy availability (19, 155). Aerobic respiration relies on mitochondrial function and is prone to produce ROS, while glycolysis primarily involves cytoplasmic metabolism, which reduces mitochondrial ROS generation, thereby decreasing DNA and protein damage and making tumor cells more likely to survive under metabolic stress (156, 157). Beyond glycolysis, tumors also rely on other metabolic pathways, such as fatty acid oxidation (FAO) and glutaminolysis, to maintain energy homeostasis (158, 159).

### 2.5 Immune checkpoints and the TME

Immune checkpoints are regulatory pathways critical for preventing autoimmunity and maintaining immune homeostasis. Immune checkpoint molecules such as PD-1/PD-L1 and CTLA-4 serve as critical regulators within the TME, maintaining immune balance under normal conditions but contributing to immune escape in cancer (41, 42). In the TME, tumor cells typically express high levels of PD-1 ligands, including PD-L1 and PD-L2 (160). PD-L1 inhibits CD8^+^ T cell responses by binding to PD-1, thereby dampening TCR signaling through recruitment of SHP-1/2 phosphatases, leading to T cell exhaustion and immune evasion (40, 161). ICIs reinvigorate T cells and unleash anti-tumor activity (162). Agents targeting the PD-1/PD-L1 axis or CTLA-4 have changed clinical management for several malignancies, including melanoma, NSCLC, and renal cell carcinoma (163, 164). Anti-PD-1/PD-L1 therapies reinvigorate exhausted CD8^+^ T cells within tumors and prime fresh T cell responses in draining lymph nodes (165). CTLA-4, expressed on activated T cells and constitutively on Tregs, competes with CD28 for binding to CD80/CD86 on APCs with higher affinity, thereby raising the activation threshold and limiting T cell priming (56, 166). Anti-CTLA-4 antibodies primarily enhance T cell priming and clonal expansion, particularly of CD4^+^ T cells (167). Combinatorial interventions targeting both CTLA-4 and PD-1 pathways have shown synergistic effects due to their non-redundant roles in different stages of T cell activation (168). Recent research continues to explore additional checkpoints beyond PD-1 and CTLA-4—such as TIM-3, LAG-3, TIGIT, and V-domain Ig suppressor of T cell activation (VISTA)—as promising targets, with numerous ongoing clinical trials (169, 170). TAMs and MDSCs contribute to immune suppression by expressing PD-L1, TIM-3, and VISTA, which further inhibit effector T cell activity (153, 171). Despite these successes, many patients either do not respond to ICIs or develop resistance (172). Mechanisms of resistance can be tumor-intrinsic, such as defective antigen presentation, or tumor-extrinsic, such as high infiltration of MDSCs and Tregs with a highly fibrotic stroma blocking T cell infiltration (173, 174). Combination therapies pairing ICIs with chemotherapy, targeted therapy, anti-angiogenic agents, or metabolic inhibitors are being explored to overcome these challenges (175).

## 3 Immunotherapeutic strategies targeting the tumor microenvironment

### 3.1 ICIs

Immune checkpoint blockade has revolutionized oncology by unleashing anti-tumor T-cell responses. Antibodies against CTLA-4 and PD-1/PD-L1 can reverse T cell “exhaustion” and reinvigorate cytotoxic lymphocytes within the TME (41). Mechanistically, CTLA-4 and PD-1 normally attenuate T cell activation (17); blocking these checkpoints restores T cell proliferation, cytokine production, and cytolytic function (176). By relieving inhibitory signals, ICIs can convert an immunosuppressive (“cold”) TME into an inflamed (“hot”) one rich in active T cells (177). Clinically, ICIs have yielded durable remissions in melanoma, lung cancer, renal carcinoma, and many other malignancies (178–180). Since the first anti-CTLA-4 (ipilimumab) approval in 2011 and anti-PD-1 (nivolumab) in 2014, dozens of indications have been reached. Notably, a new checkpoint target, LAG-3, has entered practice: relatlimab (anti–LAG-3) combined with nivolumab improved PFS in melanoma patients, leading to FDA approval in 2022 (181).

Despite these successes, only a subset of patients achieve lasting benefit. Many tumors exhibit primary resistance to ICIs due to an immunosuppressive TME or low intrinsic immunogenicity (182). Factors such as abundant Tregs, MDSCs, M2-polarized TAMs, and inhibitory cytokines in the TME can blunt T cell activity even if checkpoints are blocked (183). Tumor cell-intrinsic mechanisms also confer resistance (184). Moreover, patients who initially respond can develop acquired resistance, often via upregulation of alternative checkpoints like TIM-3 and LAG-3 or recruitment of suppressive cells to the TME (169, 183). Another challenge is immune-related adverse events, a consequence of breaking self-tolerance; these toxicities, such as colitis, dermatitis, and pneumonitis, sometimes require immunosuppressive drugs that counteract anti-tumor immunity (185, 186).

To improve ICI efficacy, combination strategies are being actively pursued. Dual-checkpoint blockade such as nivolumab plus ipilimumab has shown synergistic activity in melanoma and other cancers by targeting non-redundant inhibitory pathways (187). ICIs are also being combined with chemotherapy, targeted kinase inhibitors, and other immunotherapies to modulate the TME (188). For instance, concurrent VEGF inhibition can normalize tumor vasculature and enhance T cell infiltration, potentially boosting ICI responses (189). Similarly, ICIs combined with certain chemotherapies have yielded higher response rates and overall survival in lung and biliary cancers, without intolerable toxicity (190–192). These findings underscore that rationally countering TME-mediated immunosuppression – via multitarget regimens or novel checkpoints – is critical to extend the benefits of ICIs. Ongoing trials are evaluating new checkpoints, including TIGIT and TIM-3, and TME-modulating agents, including adenosine receptor antagonists and TGF-β inhibitors, in combination with ICIs to overcome resistance (170, 193–195). Indeed, the complex interplay between tumor, immune cells, and stroma means that multi-modal immunotherapy approaches will likely be required for patients who do not respond to single-agent ICIs.

### 3.2 Adoptive cell therapy

Adoptive cell therapies involve the infusion of immune cells – typically autologous lymphocytes – that are expanded or engineered to attack tumor cells. This modality encompasses CAR-T cells, TCR-engineered T cells, TILs, NK cells, and even genetically modified macrophages (196–199). By introducing a large population of tumor-reactive effector cells, adoptive therapy can directly augment the immune attack within the TME (196). The best-established example is CAR-T cells targeting CD19 in B-cell malignancies, which have produced high cure rates in leukemias and lymphomas (197). However, solid tumors pose additional hurdles: an immunosuppressive TME, antigen heterogeneity, and poor T cell trafficking can limit efficacy. Current research is intensely focused on adapting adoptive cell therapy to overcome these barriers (200).

CAR-T cells are T lymphocytes genetically modified to express synthetic receptors that recognize TAAs independently of MHC presentation. Upon binding antigen, CAR-T cells receive potent activation signals and release cytokines and cytotoxins to kill tumor cells (49). In solid tumors, CAR-T cells must infiltrate a hostile TME. One strategy to improve this is to target not only cancer cells but also immunosuppressive stromal components. For example, CAR-T cells directed against fibroblast activation protein (FAP) on CAFs have shown the ability to deplete fibrous stroma and thereby facilitate T cell penetration of tumors. In preclinical models of desmoplastic tumors, FAP-specific CAR-T cells eradicated CAFs, reduced collagen deposition, and allowed endogenous T cells to infiltrate and control the cancer (201). Similarly, CAR-T cells can be designed to eliminate TAMs by targeting macrophage markers. An elegant study used CAR-T cells against F4/80, a macrophage antigen, resulting in TAM depletion and improved survival in ovarian and pancreatic cancer models (202). Another group targeted the folate receptor β on M2-polarized TAMs, which led to a reprogramming of the TME: removing these suppressive macrophages triggered a higher influx of CD8^+^ T cells and enhanced anti-tumor immunity. These approaches demonstrate that adoptive cells can be engineered to modulate the TME itself – for instance by secreting cytokines, degrading stroma, or carrying enzymes to counteract inhibitory metabolites (203).

CAR-T cells are also being armored to resist TME immunosuppressants. One major T cell inhibitory pathway in tumors is TGF-β, which can induce T cell dysfunction (204). To address this, CAR-T cells have been endowed with dominant-negative TGF-β receptors or co-expression of TGF-β antagonists. In multiple myeloma, a CAR-T cell incorporating a mutated TGF-β receptor II (unable to signal) maintained its activity in a TGF-β–rich milieu and showed superior tumor control in mice (205). Similar TGF-β–resistant CAR designs have improved efficacy in preclinical models of prostate and ovarian cancer (206, 207). CRISPR-based knockout of the TGF-β receptor in CAR-T cells has likewise been used to prevent Treg conversion and exhaustion of CAR-T cells (208). Beyond TGF-β, researchers are tackling other facets of the metabolically harsh TME: recent work suggests chronic exposure to tumor-derived lactic acid and adenosine can impair CAR-T cells (209, 210). Engineering CAR-T cells to express receptors for pro-inflammatory cytokines or to secrete stimulatory cytokines (like IL-12 or IL-18) are additional tactics to keep them functional within tumors (211, 212).

The evolution of CAR-T technology has progressed to fifth-generation. Unlike fourth-generation “armored” CARs, which secrete transgenic cytokines such as IL-12 to remodel the TME, fifth-generation CARs incorporate a truncated cytokine receptor intracellular domain directly into the CAR endodomain. The prototypical 28-ΔIL2RB-z(YXXQ) design fuses an IL-2 receptor β-chain fragment bearing a STAT3-binding YXXQ motif to a CD28–CD3ζ backbone, so that antigen engagement simultaneously triggers TCR-like signaling, co-stimulation, and antigen-dependent JAK–STAT3/5 activation—delivering the three synergistic signals required for full physiological T cell activation (213). Preclinical studies show that fifth-generation CARs confer enhanced proliferation, reduced terminal differentiation, prolonged persistence, and resistance to exhaustion, translating into improved tumor control in CD19^+^ leukemia and solid-tumor xenograft models (213, 214). Analogous JAK–STAT-augmented CAR designs are now being explored across multiple solid-tumor indications, and early-phase clinical evaluation is underway (215).

CAR-NK cells are another emerging platform; NK cells are inherently adept at tumor cell killing and do not cause graft-versus-host disease (216). CAR-NK cells targeting various antigens have demonstrated potent activity without the severe cytokine release syndrome seen in CAR-T therapy (217). CAR-NK cells can be engineered to secrete IL-15 and CCL21, enhancing their metabolic fitness, persistence, and antitumor efficacy (218). Additionally, synthetic strategies such as PD-1/PD-L1 signal inverter CARs have been developed in NK cells to counteract checkpoint inhibition in the tumor microenvironment (219). In a first-in-human trial, cord blood-derived CAR19/IL-15 NK cells in CD19^+^ B-cell malignancies yielded an ORR in the range of ≈ 48.6% (day 30/day 100), with no significant cytokine release syndrome, neurotoxicity, or graft-versus-host disease observed (220).

Lastly, TIL therapy – the expansion of a patient’s own tumor-infiltrating T cells ex vivo followed by reinfusion – has made significant strides. TIL therapy inherently provides T cells already “selected” for tumor reactivity (221). In 2024, lifileucel, an autologous TIL product, became the first FDA-approved cell therapy for a solid tumor (metastatic melanoma), achieving an ORR of ~31.4% in heavily pre-treated patients. Complete responders often enjoy long-term remissions (222).

Emerging evidence highlights the critical role of T memory stem cells, or TSCMs, as a self-renewing reservoir for durable anti-tumor immunity. TSCMs possess superior proliferative capacity, long-term persistence, and the multipotent ability to reconstitute the full spectrum of memory and effector T cell subsets (223, 224). Strategies to enrich TSCMs in adoptive cell therapy include ex vivo culture with the homeostatic γ-chain cytokines IL-7, IL-15, and IL-21, as well as pharmacological activation of the Wnt/β-catenin pathway with GSK-3β inhibitors such as TWS119 (225, 226). In clinical trials, CAR-T cell products enriched for TSCM-like phenotypes have demonstrated enhanced *in vivo* expansion, prolonged persistence, and durable remissions in B-cell malignancies (227).

### 3.3 Cancer vaccines

Cancer vaccines represent a promising strategy in tumor immunotherapy aimed at harnessing the host immune system to recognize and eliminate malignant cells. Unlike prophylactic vaccines that prevent infectious diseases, therapeutic cancer vaccines are designed to elicit robust and durable T-cell responses against TAAs or tumor-specific antigens (TSAs) already present in the host (228). These vaccines can be categorized into several types, including peptide/protein-based, nucleic acid (DNA or mRNA)-based, DC-based, and vector-based vaccines (229). Among these, DC vaccines have demonstrated the ability to prime CTLs effectively by presenting tumor-derived antigens within a highly immunostimulatory context (230).

Recent advances in genomics and bioinformatics have enabled the development of personalized neoantigen vaccines, which target patient-specific mutations to induce highly specific immune responses while minimizing off-target effects (231). Clinical studies, such as those employing mRNA-based cancer vaccines, have shown encouraging results in melanoma and other solid tumors, particularly when combined with ICIs like anti–PD-1 or anti–CTLA-4 antibodies (232, 233). However, despite these advances, several challenges remain, including tumor heterogeneity, immune tolerance, and the immunosuppressive tumor microenvironment that can dampen vaccine-induced responses (234). Ongoing efforts aim to optimize antigen selection, adjuvant formulation, and delivery systems to enhance the immunogenicity and clinical efficacy of cancer vaccines (235).

Furthermore, cancer vaccine platforms incorporating Stimulator of Interferon Genes (STING) agonists as adjuvants have shown the capacity to preferentially promote stem-like CD8^+^ T cell differentiation, potentially providing long-lasting immune memory against tumor recurrence (236).

### 3.4 Oncolytic viruses

Oncolytic virotherapy uses viruses that selectively infect and kill cancer cells while simultaneously acting as *in situ* vaccines to stimulate anti-tumor immunity. These viruses are often genetically engineered to enhance their cancer specificity and immunogenicity (237). Upon intratumoral injection or systemic administration, oncolytic viruses (OVs) replicate in tumor cells, causing lysis and release of tumor antigens, damage-associated molecular patterns (DAMPs), and inflammatory cytokines—thereby turning “cold” tumors into “hot” inflamed microenvironments (238). Talimogene laherparepvec (T-VEC), a modified herpes simplex virus expressing GM-CSF, was the first OV approved by the FDA (2015) for advanced melanoma (239). T-VEC not only lyses injected tumor cells but also promotes systemic anti-tumor immunity: the released GM-CSF recruits and matures DCs at the tumor site, enhancing cross-presentation of tumor neoantigens to CD8^+^ T cells (240, 241). In clinical studies, T-VEC increased CD8^+^ T cell infiltration within tumors and elicited regression of both injected and distant (non-injected) metastases, indicating abscopal effects driven by immune activation (241, 242).

Oncolytic herpes simplex viruses, or oHSVs, have provided particularly compelling evidence for TME immune modulation. Friedman and colleagues reported a landmark phase I trial of G207, a genetically engineered oHSV, in pediatric patients with recurrent high-grade gliomas. Intratumoral G207 administration was well-tolerated and, critically, converted immunologically “cold” glioma microenvironments into inflamed ones, with marked increases in tumor-infiltrating CD4+ and CD8^+^ T cells in post-treatment biopsies (243). More recently, Meylan and colleagues used spatial proteomics, spatial transcriptomics, and TCR repertoire analysis to dissect the immune response to rQNestin34.5v.2, an oHSV in which the viral ICP34.5 neurovirulence gene is placed under nestin-promoter control. In post-treatment glioblastoma specimens, they showed that pre-existing tumor-infiltrating T cell clones expanded locally and mediated deep, persistent cytotoxic activity, with the spatial proximity of granzyme B+ T cells to apoptotic tumor cells correlating with improved progression-free and overall survival (244).

Current trials are combining OVs with checkpoint inhibitors to achieve synergistic effects. The combination of T-VEC and pembrolizumab (anti-PD-1) in advanced melanoma yielded a high response rate, with evidence that OV-mediated inflammation sensitized tumors to subsequent checkpoint blockade (245). However, a phase III trial of T-VEC + pembrolizumab did not meet its primary endpoint for PFS, suggesting that patient selection and optimal scheduling may be critical (246). Other OVs (coxsackievirus, vaccinia, adenovirus, reovirus, etc.) are in clinical trials often combined with ICIs (247).

### 3.5 Anti-angiogenic therapy

Tumors require a blood supply, but tumor-associated blood vessels are typically abnormal – dilated, tortuous, and leaky – leading to regions of hypoxia and acidosis in the TME (248). Beyond feeding the tumor, this aberrant vasculature actively promotes immunosuppression. High interstitial pressure and irregular flow create “dead zones” where effector T cells cannot easily penetrate (249). Moreover, endothelial cells in tumor vessels express inhibitory molecules like PD-L1 and FasL, which can anergize effector T cells as they attempt to traffic into the tumor (250). Tumor-secreted pro-angiogenic factors such as VEGF, not only spur new vessel growth but also directly recruit immunosuppressive cells. VEGF is a potent chemoattractant for Tregs and MDSCs and skews macrophages toward the M2 phenotype (251). It also impairs DC maturation and induces expression of checkpoints on T cells via the transcription factor thymocyte selection-associated high mobility group box (TOX), driving T cell exhaustion (252). Thus, a VEGF-rich TME often exhibits a paucity of functional CD8^+^ T cells but an abundance of Tregs and M2 TAMs, contributing to ICI resistance (253).

Clinically, combining anti-angiogenics with immunotherapy has yielded highly effective regimens. In metastatic renal cell carcinoma, pembrolizumab plus axitinib achieved ~60% response rates and significantly extended survival, gaining first-line approval (254). Similarly, atezolizumab (anti-PD-L1) plus bevacizumab outperformed standard therapy in hepatocellular carcinoma, with patient biopsies showing increased CD8^+^ T cell infiltration post-treatment (255, 256). Mechanistically, VEGF inhibition normalizes the tumor vasculature and reduces the accumulation of MDSCs and Tregs, thereby enabling improved T cell penetration and effector function (251, 257). These combination strategies have also shown promise in endometrial carcinoma, non-small cell lung cancer, and other malignancies (175, 258, 259). Beyond VEGF, emerging targets include angiopoietin-2 (which promotes neovascularization and TAM recruitment) (260, 261). While the angiopoietin-2 inhibitor trebananib showed mixed results in clinical trials, bispecific ANG2/VEGF antibodies such as vanucizumab have demonstrated promising preclinical activity and are being evaluated clinically (262, 263).

Ramucirumab, a fully human monoclonal antibody targeting VEGF receptor 2 (VEGFR2), has emerged as a versatile anti-angiogenic agent in combination immunotherapy. In the phase III REACH-2 trial, ramucirumab significantly improved overall survival in hepatocellular carcinoma patients with α-fetoprotein (AFP) ≥ 400 ng/mL (hazard ratio [HR] = 0.710, p = 0.0199) (264). Combinations with pembrolizumab have shown activity across tumor types: in the JVDF trial, ramucirumab plus pembrolizumab achieved an ORR of 7% in previously treated gastric/gastroesophageal junction (GEJ) adenocarcinoma and 25% in the treatment-naïve setting (265, 266). In NSCLC, the Lung-MAP S1800A randomized phase II trial demonstrated an overall survival benefit for ramucirumab plus pembrolizumab over standard of care in ICI-pretreated patients (267), with a triple combination adding docetaxel under evaluation in an ongoing phase II trial (NCT04340882) (268). Furthermore, the phase III RELAY trial showed that ramucirumab plus erlotinib significantly improved PFS over erlotinib alone in untreated epidermal growth factor receptor (EGFR)-mutant NSCLC (19.4 vs. 12.4 months, HR = 0.59) (269), highlighting potential for immunotherapy-incorporating triple regimens.

### 3.6 Metabolic reprogramming of the TME

Tumor cells and suppressive immune cells compete with effector lymphocytes for nutrients while releasing metabolites that impair immune function. Metabolic reprogramming strategies aim to alter the biochemical milieu of the TME to favor anti-tumor immunity, targeting acidic high-lactate conditions, accumulated catabolites such as adenosine and kynurenine, and nutrient depletion (20, 270). In immunosuppressive TMEs, cancer cells frequently engage in aerobic glycolysis (Warburg metabolism), exporting large amounts of lactic acid (271). The resulting low pH (approximately 6.8 in tumor interstitium) directly inhibits T cell and NK cell activity while promoting polarization of TAMs and Tregs (128, 272). Lactate upregulates inhibitory checkpoints including PD-1 and CTLA-4 on T cells and drives MDSC development while reinforcing M2 tumor-promoting TAM phenotypes (144, 153). Approaches to neutralize tumor acidity include systemic buffers and inhibition of lactate dehydrogenase (LDH) or glycolysis (273). Short hairpin RNA (shRNA)–mediated LDH-A silencing in melanoma cells decreased lactate production, downregulated tumor PD-L1 expression, and, when combined with anti-PD-1, enhanced intratumoral CD8^+^ T cell and NK cell infiltration with concurrent Treg reduction in B16-F10 melanoma models (274). Another strategy involves blocking lactate export via monocarboxylate transporter (MCT) inhibition. The MCT1 inhibitor AZD3965 has entered clinical trials (275), and preclinical studies showed that dual MCT1/MCT4 blockade significantly prolonged survival by trapping lactic acid inside tumor cells and preventing TME acidification (276).

Adenosine represents another prominent immunosuppressive metabolite, particularly under hypoxic conditions (277). ATP released by dying cells is rapidly converted to adenosine by ectonucleotidases (CD39, CD73), which signals through A2A and A2B receptors to suppress TCR signaling and macrophage activation (46, 278). Multiple agents are in trials, including A2A receptor antagonists such as ciforadenant and CD73 inhibitors (279). Early-phase data from renal cell carcinoma studies suggest that A2A blockade can be safely combined with ICIs (280). Notably, adenosine via A2B receptors skews macrophages toward an M2 phenotype; thus, blocking adenosine could re-polarize macrophages toward a pro-inflammatory M1 state (281).

The tryptophan-kynurenine pathway represents another metabolic checkpoint, as tumors often express indoleamine 2, 3-dioxygenase (IDO), degrading tryptophan into immunosuppressive kynurenine (282). IDO inhibitors such as epacadostat showed promise in early trials; however, a large phase III trial of epacadostat with pembrolizumab in melanoma (ECHO-301/KEYNOTE-252) was negative, revealing that single-pathway metabolic interventions may be insufficient (283). Interest remains in dual IDO/TDO inhibitors and combinations with other therapies (284). Additional strategies include supplementing arginine to counter depletion by arginase-expressing MDSCs (285), evaluating drugs like metformin that alter tumor and T cell metabolism (286), and leveraging nanotechnology for local pH modulation (287).

### 3.7 Targeting immunosuppressive cells in the TME

Tumors exploit various immunosuppressive cell types to evade immune attack. Key players include Tregs, MDSCs, TAMs, and CAFs, which collectively create a tolerogenic niche by secreting inhibitory cytokines, depleting nutrients, and expressing checkpoint ligands. Therapeutic strategies to deplete these cells or neutralize their suppressive activity aim to reactivate anti-tumor immunity (288, 289).

Tregs accumulate in many tumors and suppress effector T cells via CTLA-4-mediated CD80/86 downmodulation on DCs, secretion of IL-10 and TGF-β, and consumption of IL-2 (290). High intratumoral Treg abundance generally portends poor prognosis (291). Therapeutic approaches include targeting IL-2 receptor alpha (CD25) and CCR4, a chemokine receptor enabling Treg trafficking to tumors (292). Mogamulizumab, an anti-CCR4 antibody, depletes Tregs in peripheral blood and has been evaluated with ICIs in solid tumors. A phase I/II trial combining mogamulizumab with nivolumab in 114 patients with advanced solid tumors showed acceptable safety with an ORR of 10.5%, though this did not demonstrate superior efficacy compared to nivolumab alone (293). Preclinically, CCR4-targeted therapies show promise by eliminating both malignant cells and immunosuppressive Tregs. Watanabe et al. engineered CCR4-CAR-T cells based on mogamulizumab that selectively depleted CCR4^+^ Tregs while sparing CD8^+^ and Th1 cells, demonstrating superior antitumor efficacy and long-term remission in T-cell lymphoma mouse models (294).

MDSCs are immature myeloid cells that inhibit T-cell function via arginine depletion through arginase, production of nitric oxide, ROS, and secretion of cytokines including IL-10 (90). Tumors secrete factors such as GM-CSF, G-CSF, and IL-6 to expand MDSCs (295). Therapeutic strategies include inhibiting MDSC recruitment by blocking chemokine receptors such as CXCR2 and promoting their differentiation into non-suppressive cells (296). A seminal study in pancreatic ductal adenocarcinoma demonstrated that CXCR2 inhibition reduced intratumoral MDSCs, improved T-cell infiltration, and when combined with anti-PD-1, significantly extended survival in mouse models (297). Another approach targets MDSC survival pathways through inhibitors of STAT3 or PI3Kγ (298, 299). PI3Kγ inhibitors are particularly promising as they reverse MDSC suppressive capacity and repolarize TAMs toward a pro-inflammatory phenotype. Preclinical studies demonstrated that combining PI3Kγ inhibition with anti-PD-1 or anti-CTLA-4 resulted in superior tumor control compared to either agent alone, by removing a major source of checkpoint ligand expression and immunosuppressive cytokines (300, 301). Innovative CAR-T approaches have also been developed to combat MDSCs. In glioblastoma models, CAR-T cells engineered to secrete IL-15 within tumors sustained CAR-T function while converting local MDSCs into activated macrophages or dendritic-like cells, effectively nullifying their suppressive role (302).

Macrophage-directed therapies represent a rapidly expanding frontier in TME-targeted immunotherapy. CSF-1 receptor (CSF-1R) inhibitors such as pexidartinib deplete or reprogram TAMs by blocking the CSF-1/CSF-1R survival axis; pexidartinib received FDA approval in 2019 for tenosynovial giant cell tumors based on the ENLIVEN trial (ORR 38%) and is now being explored in combination with checkpoint inhibitors in solid tumors (303). The CD47–SIRPα innate immune checkpoint has also attracted attention, as CD47 functions as a “don’t eat me” signal that inhibits macrophage phagocytosis. Although magrolimab (anti-CD47) initially showed encouraging responses with azacitidine in Myelodysplastic Syndromes (MDS) and Acute Myeloid Leukemia (AML) (304), all three pivotal phase III trials (ENHANCE, ENHANCE-2, ENHANCE-3) demonstrated futility and increased mortality, prompting Gilead to discontinue its development in hematologic malignancies in 2024 (305, 306); whether this axis remains viable in solid tumors is under investigation. Complementary approaches include repolarizing M2-like TAMs toward a pro-inflammatory M1 phenotype using PI3Kγ inhibitors, TLR agonists, or STING agonists, each potentially synergizing with checkpoint blockade (301). On the cellular therapy front, CT-0508, a HER2-targeted CAR-macrophage (CAR-M), demonstrated safety and manufacturing feasibility in a first-in-human phase I trial, with 44% of HER2 3+ patients achieving stable disease and correlative analyses confirming CAR-M trafficking and CD8^+^ T-cell expansion within the TME (307). Although objective responses remain to be demonstrated, these findings provide proof-of-concept for CAR-M therapies in solid tumors. Engineered macrophage-derived exosomes loaded with therapeutic payloads represent yet another emerging platform for TME modulation (308).

## 4 Discussion

The TME is a heterogeneous, plastic ecosystem shaped by immune cell reprogramming, cytokine imbalance, and ECM remodeling, and serves as a central site of tumor immune evasion (7). Cancer immunotherapy aims to reactivate or redirect the host immune response to eliminate tumor cells, representing a shift from cytotoxic to precision-based treatment (3, 309). Over the past decade, our understanding of the TME has expanded rapidly, yet major challenges persist. The TME’s dynamic and heterogeneous nature reflects variations in immune cell composition, metabolic states, and stromal architecture, which requires the continued application of multidisciplinary approaches (310). While immunotherapies have revolutionized the treatment of various malignancies, therapeutic resistance and immune-related toxicities emphasize the need for deeper mechanistic insights into TME–immune cell interplay and the development of more sophisticated combination regimens (10, 311).

Clinically, sustained therapeutic responses have been documented in immunologically “hot” tumors such as melanoma and microsatellite instability-high (MSI-H) colorectal cancer, where high tumor mutational burden (TMB) and pre-existing T cell infiltration create a favorable milieu for checkpoint inhibitor efficacy (312). Conversely, strategies to convert “cold” tumors into “hot” ones are actively being explored. In pancreatic ductal adenocarcinoma, combinations of CXCR4 antagonists with anti-PD-1 have demonstrated enhanced T cell infiltration in clinical trials (313). Similarly, in glioblastoma, intratumoral delivery of oncolytic viruses combined with checkpoint inhibitors has shown preliminary evidence of immune activation in early-phase studies (314). These examples highlight the importance of tailoring immunotherapeutic strategies to the specific immune landscape of each tumor type.

This review systematically dissects the intricate immune regulatory networks within the TME, centering on the dynamic crosstalk among tumor cells, cytokines and chemokines, immune cells and metabolic reprogramming that shapes the immunosuppressive TME. Moreover, we focus on the clinical advances in TME-targeted therapies and their translational potential in cancer treatment. In the future, precision oncology approaches will increasingly depend on predictive biomarkers to guide patient-specific treatment—be it with checkpoint inhibitors, metabolic modulators, angiogenesis blockers, ECM remodelers, or adoptive cell therapies (315). Another key priority is balancing potent anti-tumor immunity with the risk of autoimmunity. Improving preclinical models is considered to be an effective approach to better capture the complexity of human tumor–immune interactions. Such refinements will accelerate the translation of experimental advances into clinical applications.

This review underscores that decoding the context-dependent immune regulatory mechanisms in the TME is the prerequisite for developing effective translational strategies, and personalized combinatorial therapies tailored to TME phenotypes may help overcome therapeutic resistance. Nevertheless, this review has inherent limitations: First, evidence supporting TME-driven immune regulation is highly context-dependent across tumor types, disease stages, and treatment settings, which may limit the generalizability of specific targets and combinations. Second, many mechanistic insights are derived from selected preclinical models whose immune composition, spatial architecture, and therapy-induced remodeling do not fully recapitulate patient tumors, underscoring the need for improved translational models and functional validation in clinically annotated specimens. Finally, although multiple biomarker candidates and combination strategies are emerging, their clinical utility requires prospective validation, standardized assays, and careful evaluation of safety and toxicity management.

In summary, the TME both drives therapeutic resistance and provides actionable targets for intervention. Progress in single-cell and spatial profiling, rational combination design, and biomaterial-based local delivery is likely to define the next phase of TME-targeted immunotherapy.
